# Potential Applications and Limitations of Electronic Nose Devices for Plant Disease Diagnosis

**DOI:** 10.3390/s17112596

**Published:** 2017-11-11

**Authors:** Antonio Cellini, Sonia Blasioli, Enrico Biondi, Assunta Bertaccini, Ilaria Braschi, Francesco Spinelli

**Affiliations:** Department of Agricultural Sciences, Alma Mater Studiorum—University of Bologna, viale G. Fanin 44, 40127 Bologna, Italy; antonio.cellini2@unibo.it (A.C.); sonia.blasioli@unibo.it (S.B.); enrico.biondi3@unibo.it (E.B.); assunta.bertaccini@unibo.it (A.B.); ilaria.braschi@unibo.it (I.B.)

**Keywords:** e-nose, VOCs, plant pathogen detection, pest infestation, gas sampling, sensor signal drift, relative humidity control

## Abstract

Electronic nose technology has recently been applied to the detection of several plant diseases and pests, with promising results. However, in spite of its numerous advantages, including operational simplicity, non-destructivity, and bulk sampling, drawbacks include a low sensitivity and specificity in comparison with microbiological and molecular methods. A critical review of the use of an electronic nose for plant disease diagnosis and pest detection is presented, describing the instrumental and procedural advances of sensorial analysis, for the improvement of discrimination between healthy and infected or infested plants. In conclusion, the use of electronic nose technology is suggested to assist, direct, and optimise traditionally adopted diagnostic techniques.

## 1. Introduction

Traditionally, diagnostic assessments in plants have been carried out by the analysis of disease symptoms, pest morphology, as well as by immunochemical or metabolic assays [1,2,3]. Such a *modus operandi* may not be suitable in some situations, for instance, when symptoms appear in a late phase of infection and curative interventions are ineffective [4,5], or when the identification of a pest may not be univocal at certain life stages. To achieve an early diagnosis on plant-derived material independently from symptom development, molecular techniques based on polymerase chain reaction (PCR) have been adopted and internationally recognised over time, as recommended by the European Plant Protection Organization (EPPO) [6]. Such molecular methods have quickly become the state of the art for the detection of a large number of pathogens because of their specificity and sensitivity, together with their increasingly low operational costs and automatisation [1]. However, molecular methods present some key issues, such as the design and validation of primers, the applicability of each primer set to single, specific targets, and the need of a sampling protocol, resulting from the trade-off between plant sample representativeness and availability of plant material. In fact, the analysis is destructive, and the sampling procedure may impair the viability and economic value of the sampled material. Therefore, alternative methods were considered to substantiate and assist molecular and traditional diagnostic techniques by circumventing their drawbacks.

Among these, the recognition of infected or infested plant material, based on their emission of volatile organic compounds (VOCs), has been attempted. The theoretical frame of VOCs-based methods consists of the generation of different (and, in some cases, pathogen-specific) VOCs emissions, according to the plant’s health status and the biochemical interactions between the plant host and its pest or pathogen. Some key compounds taking part in plant defences are, in fact, volatile, including protective compounds (e.g., terpenoids, essential oils), plant hormones (e.g., ethylene, jasmonates, methyl-salicylate), insect pheromones, digestive or metabolic byproducts, and compounds deriving from cell damage [7].

In spite of their low specificity compared to molecular methods, VOCs-based diagnostic applications were developed by plant pathologists and parasitologists due to some considerable advantages. Firstly, the analysis is non-destructive, thus, it may be applied on stored crops or fully-grown plants and reiterated for a desired time course on the same samples, without compromising the value or productivity of the analysed plant material. In addition, the gas sampling may be applied to bulk samples, overcoming representativeness problems. Unlike classical PCR methods that are highly specific for a single pathogen, VOCs-sampling can be used to discriminate among different diseases at the same time. Finally, the diagnostic system may be trained to increase its discrimination power between healthy and infected samples, or to widen the spectrum of recognition to multiple classes of pathogens.

Gas chromatography-mass spectroscopy (GC-MS) and, more recently, proton transfer reaction-mass spectroscopy (PTR-MS) and matrix assisted laser desorption/ionization time of flight-mass spectroscopy (MALDI-TOF) have demonstrated a remarkable discrimination potential for a number of plant diagnostic applications. In this context, successful VOCs-based detection of infected plant material has been achieved on apple and pear plants [8,9], grapevine plants [10], forestry and urban shade tree species [11,12,13], oil palm [4], apple fruit [14], onion bulbs [15], and potato tubers [16,17,18,19]. 

On the other hand, the high requirements of such technologies in terms of operational costs and personnel commitment have limited their diffusion and practical application for diagnostic purposes. While the use of PTR-MS and MALDI-TOF has resulted in little more than positive proofs of concept so far [19,20,21,22], more work has been carried out with regard to the GC-MS characterisation of plant material subjected to a variety of pests or pathogens [1,2]. In these cases, the success of discrimination between healthy and infected or infested samples was often conditioned by the finding of specific marker compounds. However, this optimal condition is not very frequent, as most of the differences in VOCs emissions are of a quantitative and non-specific nature. In fact, the VOCs blends from diseased plants are highly affected by disease development, superinfection by opportunistic pathogens, and plant phenological stage. 

## 2. Electronic Nose Plant Diagnostic Applications

The electronic nose (e-nose) does not allow the analytical determination of VOCs or their quantification in the blend. However, it provides an easily-operated, quickly-responding, and flexible tool for the recognition of differences in gas samples. The e-nose operating principle resides in the variation of the electrical conductivity of its sensors, depending on the chemical interaction of their surfaces with the surrounding gas phase. An e-nose is fundamentally composed by an array of sensors with no strict specificity, but presenting different sensibilities to molecules belonging to several chemical classes or having diverse functional groups. The electric signals from the sensors are elaborated to generate a pattern corresponding to the gas composition of the sample [23]. Thus, air samples containing different VOCs compositions are described by unique e-nose profiles, which may be compared to the overall variation of a pool of reference gas samples. 

For all the characteristics reported above, e-nose technology has gained momentum for applications in a wide range of fields, including human diagnostics, food quality, and environmental safety [24,25,26,27]. Uses of the e-nose in agriculture, botany, and forestry included the identification of cultivars, species, or wood, and the detection of pesticide residuals. In the last two decades, e-noses have been tested for diagnostic applications on a wide variety of pathosystems, with performances comparable to GC-MS in terms of efficacy while requiring lower analytical time and post-analysis elaboration, along with lower running costs. Plant material infected with pathogenic bacteria or fungi or infested with or damaged by pests has been subjected to e-nose detection (Table 1). It is worth noting that in only two cases of study was an e-nose prototype specifically designed for the application under investigation. In all the other works, commercially available devices were adopted for the experimental conditions.

The list of commercial e-nose models employed for plant diagnosis purposes is provided in Table 2. Three commercial e-noses (PEN3 by Airsense Analytics, Schwering, Germany; EOS835 and EOS507C by Sacmi, Imola, Italy) based on metal-oxide sensor (MOS) technology were successfully applied by the authors for the discrimination of infected material in a variety of pathosystems: *Erwinia amylovora* on apple and pear plants and fungal rots on kiwifruit [8,9,36], *Agrobacterium vitis* on grapevine [10,28], *Clavibacter michiganensis* ssp. *sepedonicus* or *Ralstonia solanacearum* on potato tubers [18], *E. amylovora* and *Pseudomonas syringae* pv. *syringae* on cold-stored dormant apple plants [20], and *P. syringae* pv. *actinidiae* on kiwifruit propagation material [22]. 

The e-nose analysis on plant material reveals some intrinsic critical points that may affect sensitivity and specificity of the technique. VOCs profiles of such biological samples, in fact, are constantly evolving with time as a result of (i) the natural ageing of living material; (ii) the plant physiological status; (iii) the disease severity due to primary infections; (iv) the disease incidence in bulk samples; (v) the disease evolution represented by the attack of secondary and opportunistic pathogens; and (vi) the environmental conditions, such as light, temperature, and relative humidity, that strongly influence VOCs emission by plants and fruit. In this review, the optimal set-up of some critical operative and instrumental parameters necessary to obtain a significant e-nose response aimed at discriminating healthy from diseased samples is pointed out. Gas sampling, instrument training and data processing, sensor response drift, and influence on the analytical response are discussed, and practical solutions are proposed.

## 3. Gas Sampling Techniques

Most e-nose models are designed to work on ambient air or static headspaces. This operational mode is the simplest and the least prone to artifacts, and it is most frequently adopted for the diagnosis of diseased plant material. In some cases (such as the PEN3 e-nose model), the instrument allows to set the gas flow rate in the sensor chamber: higher flow rates may improve the instrumental sensitivity to some extent by exposing the sensors to a larger air sample and thus enhancing the signal-to-noise ratio and the sensor response. However, this option may be hard to apply on small-volume gas samples, where the headspace would be completely consumed before obtaining a reliable reading.

An alternative is represented by the use of thermally desorbable cartridges for VOCs adsorption. These VOCs concentration units may be used for active sampling (by fluxing the cartridge with a definite volume of air) or passive sampling (by exposing the cartridge to the sample for a definite time). In both sampling procedures, VOCs adsorbed by the cartridge are desorbed in a smaller volume of sample air, thus obtaining a VOCs concentration effect and, presumably, an increased instrumental sensitivity. A variety of sorbent materials (e.g., Carbotrap, Carboxen, Tenax-TA, Tenax-GR, Poropak Q, Hayesep Q), with different chemical affinity to VOCs [44] are commercially available and may be selected to focus the analysis on VOCs classes of interest, thus reducing the background variability [45,46] when the VOCs composition or the biochemical peculiarities of the biological system under investigation are known. An example of the effect of commercially available VOCs-sorbent cartridges, coupled to PEN3 e-nose, on the efficacy of detection or recognition is reported in Table 3. Carbotrap showed the highest ability to concentrate VOCs from potato tubers infected by *R. solanacearum*, while Tenax-TA or Carboxen performed best with volatiles from *C. michiganensis* ssp. *sepedonicus*-infected samples. Thus, the choice of a sorbent material for the detection of multiple pathogens should account for its sensitivity to VOCs emitted or induced by each of them. 

An active VOCs concentration unit is embedded in some commercial e-noses, such as PEN3 (Electronic Desorption Unit, EDU3). However, passive gas sampling using a sorbent cartridge may be easily applied to any e-nose model. For instance, a sampling protocol was developed by the authors, based on commercially available cartridges (Radiello™, Supelco, Bellefonte, PA, USA), desorbed at 380 °C for 10 min in a 1 L gas-sampling bag with chromatographic-grade air. The parallel employment of such sampling protocol and of active gas sampling on potato tubers infected with *R. solanacearum* or *C. michiganensis* ssp. *sepedonicus* and stored in a jar at room temperature yielded comparable results [18]. Interestingly, the discrimination was unsuccessful when performed with no accumulation step.

One practical advantage of passive sampling emerges when it can be performed over an extended period, such as during transport or storage of plant material. In fact, the time lapse between the beginning of the storage and the diagnostic analysis may be employed to concentrate sample-derived VOCs on a sorbent cartridge, thus facilitating the discrimination of the diseased material. Examples of similar applications were reported for potato tubers [18] and apple scions [20]. The authors found encouraging results by analysing volatile profile of asymptomatic plant material, infected with quarantine bacterial pathogens, in simulated conditions of custom and nursery storage. In both the experiments, a correct classification was achieved for more than 80% of the samples.

## 4. Training, Data Elaboration, and Decision-Making

The initial stage of an e-nose analysis consists of a training session, in which known samples are presented to the analytical system. Indeed, one of the main characteristics of e-noses is the possibility to indefinitely train its detection system, to refine the definition of patterns (thus increasing the discrimination power), and to expand the database of VOCs profiles. The last feature represents one of the main advantages of plant diagnosis by e-nose in comparison with molecular methods, as multiple pathogens may be screened at the same time and on the same biological sample. Principal component analysis (PCA) and hierarchical clustering may be useful in this phase [47] to visualise the discrimination power of the method, and to evaluate the contribution of each sensor to overall discrimination. Some sensors may provide redundant information, contribute poorly to the separation of data clusters, or even generate background noise. In such cases, their exclusion from the analysis may, in fact, improve the diagnostic performance [48]. The decision to exclude one or more sensors from the analysis can be taken after building the loading plot (i.e., the visualisation of the contribution of each sensor to the principal components of variance). Most of the sensors, for instance, were found to be negligible for the recognition process of *R. solanacearum*- and *C. michiganensis* ssp. *sepedonicus*-infected potato tubers from healthy ones [18]. The exclusion of these sensors did not decrease the value of the first principal component but significantly improved the data classification. Therefore, it should be pointed out that a high number of sensors in an e-nose equipment does not necessarily imply a high discrimination power. Instead, a sensor array yielding uncorrelated (i.e., non-redundant) responses should be preferred, and the selection of sensors with a relative specificity for marker compounds may improve the diagnostic performances.

Subsequent to training, a validation step is required, in which the sensorial analysis of known samples not used for training is compared to the training database according to a classificational algorithm, such as K-th nearest neighbour, discriminant function analysis, artificial neural networks, or support vector machines [43,47]. In this step, the percentage of correct identification is calculated. When the number of known samples available is not sufficient for both an efficient training and a reliable validation, a Leave-one-out cross-validation may be adopted: each sample, in turn, is excluded from the training database, and its classification is attempted according to its similarity to the rest of the data set.

Although the e-nose-based diagnosis has mostly focused on single pests or pathogens, the technology and data elaboration may, in principle, apply to more than two (healthy/infected) classes. In previous research [12,13,20,32,38], multiple-class diagnosis was achieved to a certain degree on woody plants, apple scions, blueberry fruit, and potato tubers, in which the infection with different pathogens could be effectively told apart. In other cases, infected samples were identified, although the two pathogens could not be discriminated [18,36]. A possible explanation of the lack of discrimination between two pathogens may be the predominance of plant-derived VOCs in the sample air: for example, potato tubers infected by *R. solanacearum* and *C. michiganensis* ssp. *sepedonicus* present similar symptoms (ring rot and brown rot, respectively), due to the pectinolytic activity of both pathogens, and the tissue degradation process is presumably responsible for the common VOCs emission.

**Figure 1:**
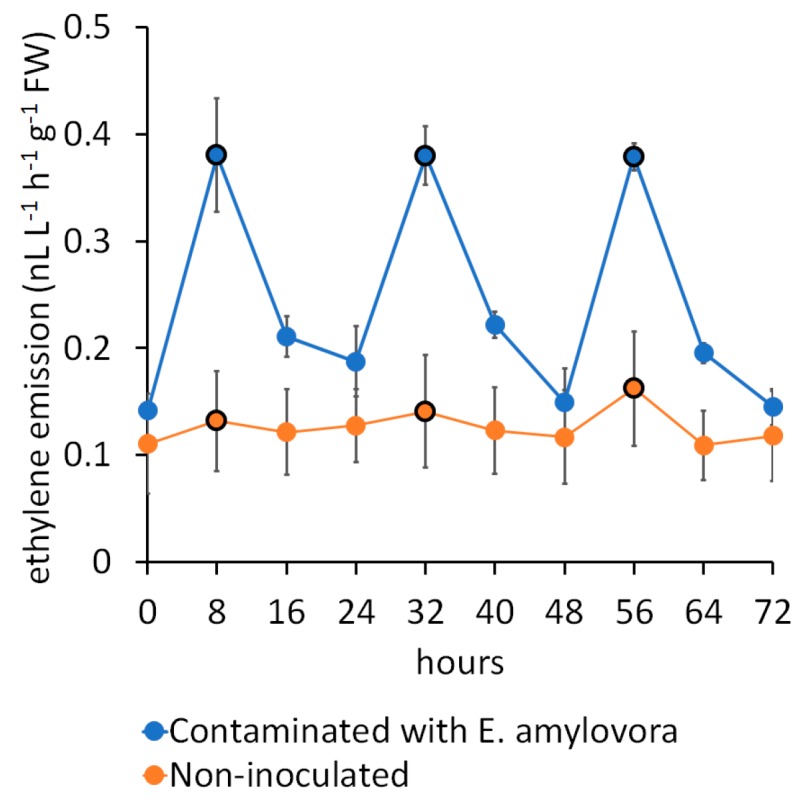
Ethylene release from in vitro kiwifruit plants contaminated with the incompatible pathogen *Erwinia amylovora*. The samples were enclosed in 150 mL pots. The GC analysis started 24 h after inoculation and was repeated every 8 h under a 16 h light/8 h dark photoperiod. The samples were vented after each reading. Data points circled in black correspond to ethylene accumulation during the dark phase.

Since the recognition of different sample classes is performed on the basis of statistical variability among the VOCs profiles of their elements (i.e., clusters are characterised by a lower in-group variability, compared to between-groups), biological samples with a VOCs release influenced by accidental conditions may represent a serious challenge for VOCs-based recognition. Ethylene, for instance, in addition to being possibly directly perceived by e-nose sensors [24], regulates the release of a plethora of plant compounds linked to fruit ripening, senescence, and defence [49,50]. Since leaf stomata are the interface between photosynthetic tissues and their gaseous environment, factors affecting stomatal closure may change the plant’s VOCs profile as well [51]. Further sources of variability may be photoperiodism and/or the activity of the microbial community. For instance, the inoculation of micropropagated kiwifruit plants with the incompatible pathogen *E. amylovora* resulted in an increased ethylene release in the dark, as shown in Figure 1. Thus, the same plants analysed in light or in dark conditions would fall into two different clusters.

**Figure 2:**
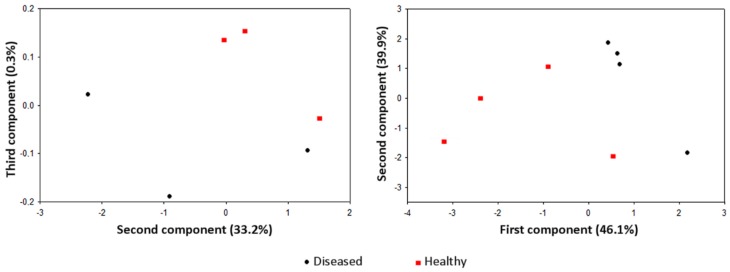
Score plot on the plane obtained with the second and third principal components of e-nose readings taken on healthy or *E. amylovora*-infected apple flowers (left panel) or detached branches (right panel), 3 days after inoculation. EOS835 e-nose was used with direct VOCs sampling. The discrimination of the two classes was not possible on the first component of total variance in flowers.

Similarly, plant tissues with an inherently high VOCs release, such as flowers, may be prone to a high background noise, preventing an effective e-nose discrimination. In fact, in a preliminary GC-MS screening of VOCs released by *E. amylovora*-infected apple flowers, the authors only observed a quantitative variability in the emitted compounds, poorly correlated with the infection status, and an unsatisfactory e-nose clustering of infected samples (Figure 2).

## 5. Effects of Disease Progression, Severity, and Incidence

The most widely used pattern recognition methods consist of a univocal attribution of each plant sample to one of the categories adopted during the e-nose training. However, such a situation may not adequately represent quantitative differences among samples, deriving from different infection or infestation incidences or severities. 

The e-nose technology is not suitable for quantitative determination in complex gas mixtures [52]. However, the distance of VOCs profiles from a group of healthy control samples, as measured by PCA or by Euclidean distance, may be taken in principle as a measure of different degrees of disease or infestation severity in plants. This effect is evident for some simplified experimental settings, in which the pests or the infected plant samples are easily enumerated, and the sources of background VOCs are reduced. For instance, a remarkable correlation between e-nose response and number of individual pest insects was observed in sealed flasks [30,40]. However, when infested rice plants were used, a similar correlation was found only during the first hours of the experiment [42]. The application of e-nose recognition to damaged wheat samples after removing the pest resulted in a good sample clusterisation but no response linearity [41].

In other cases, the distribution of VOCs profiles was compatible with a linear function of disease incidence, progression, and/or severity [10,18,33,39]. The experimental conditions (such as temperature or sampling protocol) could highlight one disease parameter (incidence or severity) over the others. Laboratory experiments, for instance, allowed to identify *R. solanacearum*- and *C. michiganensis* ssp. *sepedonicus*-infected potato tubers according to severity, while large-scale experiments in cold storage were more sensitive to incidence [18]. Thus, it may be speculated that the overall profile is affected by several sources of variability, and some of these may prevail under particular conditions.

Therefore, the operative conditions (i.e., those allowing the best performance or those commonly encountered in practice) and the instrumental sensitivity (i.e., the minimum incidence, severity, or pathogen or pest population allowing detection) should be considered to develop a reliable diagnostic protocol based on e-nose discrimination, and the definition of a risk index based on a detection threshold may replace the positive attribution of the sample to one class of infection. Because of the possibility of latent infections, e-nose diagnosis could be oriented, in these cases, to the rapid elimination of surely infected samples, so that more refined diagnostic procedures (such as PCR recognition) may focus on dubious or apparently healthy samples.

## 6. Signal Drift Effects and Sample Humidity Control

**Figure 3:**
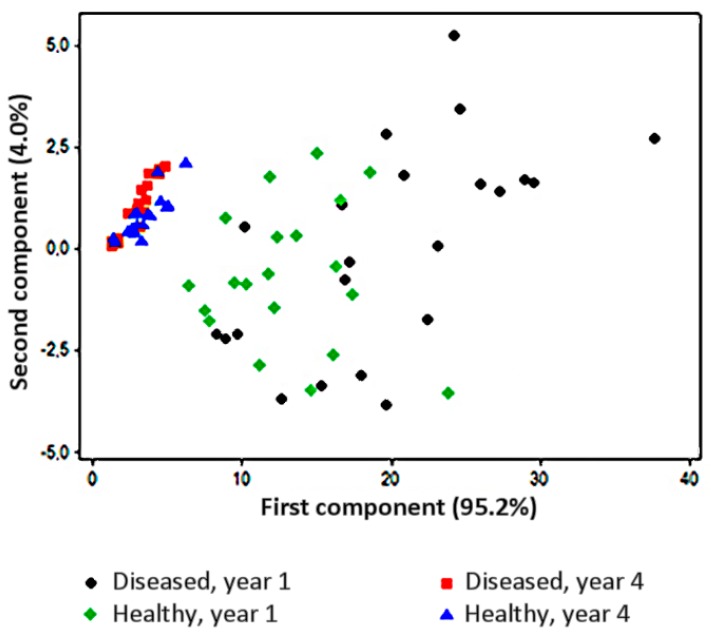
Score plot on the plane defined by the first two principal components of e-nose readings taken on healthy or *A. vitis*-infected grapevine rootstocks, 1 or 4 years after inoculation. The analysis was performed using the PEN3 e-nose with direct headspace sampling on 20–25 cm rootstock segments placed at 40 °C overnight in individual 150 mL glass tubes containing silica gel (approx. 3 g) as a desiccant.

Drift effects, due to sensors degradation, and variations of relative humidity (RH) may hinder the repeatability of analysis in different experimental sessions, jeopardising long-term instrumental training. However, sensor responses are automatically normalised after instrumental calibration in several modern e-noses, thus compensating the systematic error in the output. In such conditions, e-noses may achieve a remarkable stability even after years. However, the entity of sensor drift should be compared to the global variability among the samples. For instance, *A. vitis*-infected grapevine rootstocks could be recognised nine months after inoculation in agreement with their disease severity (tumour size), using the PEN3 equipment [10]. The authors replicated the experiments three years later, and the VOCs profiles of healthy samples were still comparable, with a drift (standard deviation of sensor responses) of about 7% for each data set. On the other hand, sensor drift contributed to total variance more than the effect of the disease on VOCs profiles. Finally, infected samples presented a lower disease severity and, therefore, the cluster separation was less obvious (Figure 3). 

**Figure 4:**
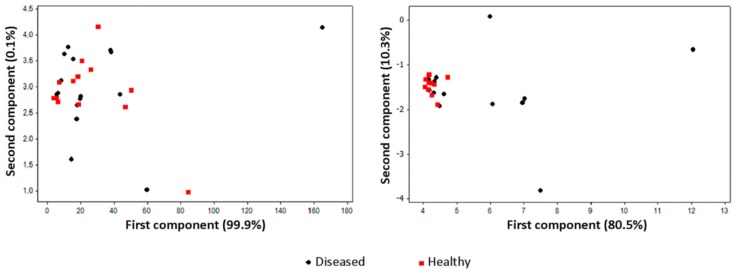
Score plots on the plane defined by the first two principal components of volatile compounds obtained by sensorial analysis with (left panel) and without (right panel) a desiccant (SiO_2_). The analysis was performed using a PEN3 e-nose with active sampling on Tenax-TA, on unwounded, *Clavibacter michiganensis* ssp. *sepedonicus*-infected or healthy tubers, maintained for one day at room temperature in a sealed 0.5 L jar.

RH is a major issue in e-nose analysis, since it substantially affects both quantitatively and qualitatively the composition of the gas phase. In fact, water vapour is perceived by e-nose sensors like any other volatile compound [27,53]. In addition, the overall composition of the gas phase results as a dynamic equilibrium among its components, i.e., the increase of one of them (such as water vapour) may cause the reduction of the others. Finally, the water in the gas phase would determine an increased presence of hydrophilic over hydrophobic compounds. For these reasons, the normalisation of RH must be accounted for e-nose applications to biological systems usually containing high water amounts. In most cases, treating the gas sample with a desiccant may be sufficient to obtain the normalisation of e-nose results. Styrene (a water-insoluble compound), for instance, was identified as a marker of *A. vitis* infection on grapevine plants [10]. Thus, the discrimination of diseased from healthy samples was not affected by the presence of the desiccant. On the contrary, the discrimination between healthy and infected potato tubers was only possible without desiccation [18], since most of the brown rot and ring rot markers were hydrophilic compounds [19], and the use of a desiccant would abate their concentration in the headspace (Figure 4). 

**Figure 5:**
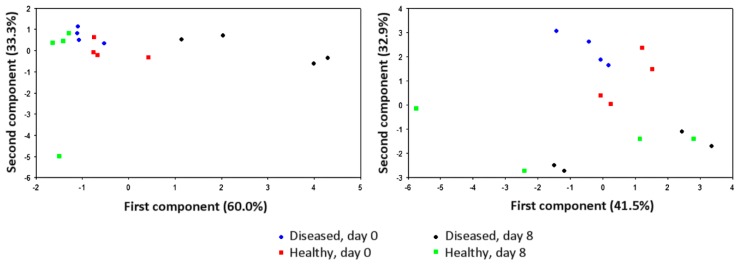
Score plots on the plane defined by the first two principal components of volatile compounds obtained by sensorial analysis of in vitro apple explants infected with *E. amylovora*, 0 and 8 days after inoculation. The two panels refer to analysis with EOS507C (left, with negligible drift of healthy samples) and with PEN3 (right, presenting a time-based drift).

In some cases, water vapour may not be completely or efficiently removed from plant samples characterised by a very high RH, and the lack of control on this parameter would determine an overall decrease in discrimination power. When gas phase desiccation is not feasible or desirable (for instance, because this would compromise the plant viability, as in the case of in vitro explants), RH may be raised to values close to 100% [33,36]. Alternatively, the EOS507C model integrates an automatic compensation system to adjust sample air to a pre-set RH value. This instrument proved to be suitable for the analysis of in vitro plant samples [20,22], whereas other e-nose models showed a day-based clusterisation, as shown in Figure 5.

## 7. Conclusions

Several key issues have so far prevented e-nose detection to meet the reliability requirements of international plant protection organisations, such as EPPO, and to become a routine practice for phytosanitary services. Independently developed, general-use e-noses may show different performances even with a standardised protocol of sampling and analysis [12]. Thus, the customisability of the sensor array and an interface with the manufacturer may represent an added value to the instrument. However, the e-nose technology has significantly progressed since the first attempts of application to plant diagnosis and may become mature in a near future. For instance, sensor drift effects have been drastically reduced and RH control has been introduced in several recently released commercial e-nose models. However, sensor drift is still one of the main issues preventing the build-up of long-term VOCs profile databases. In this view, the study of sensor aging could contribute to diagnostic applications by predicting and normalising time-dependent changes in sensor response.

Some aspects still to be tackled are relevant to the standardisation of biological samples, rather than to the instrumental design. For instance, the development of semi-specific adsorbent cartridges, together with the standardisation of sampling conditions, may be useful to remove some of the factors contributing to background noise, such as water vapour or ethylene. 

Because of the nature of biological systems, which may evolve over time and express different features according to external conditions, the control of time-dependent systematic errors and background variation appears of utmost importance. Therefore, plant researchers, field operators, and e-nose developers should cooperate to achieve ad hoc solutions for well-defined phytosanitary concerns, with consideration for the inherent variability of the biological players under analysis. In perspective, the e-nose analysis on plant material may be employed to assist other diagnostic techniques, such as PCR-based ones, to provide a rapid screening of samples and optimise time and resource commitment in the detection of pathogens and pests.

## Figures and Tables

**Table 1:** Applications of e-nose to plant disease and pest diagnosis. Pathosystems, analytical details and e-nose model are summarised.

Pathosystem and Plant Material	Analytical and Technical Solutions	E-Nose Model(s)
Multiple wood decay fungi on wood samples of several tree species [12]	Static headspace analysis Multiple-class recognition	AromaScan A32 SLibraNose 2.1 PEN3
Multiple root decay fungi on root segments of several shade tree species [13]	Static headspace analysis Multiple-class recognition	PEN3
*Agrobacterium vitis* on grapevine rootstock cuts [10]	Static headspace analysis	PEN3
*Ralstonia solanacearum* and *Clavibacter michiganensis* ssp. *sepedonicus* on potato tubers [18]	Lab to real scale VOCs concentration on adsorbents Sample air desiccation with SiO_2_	PEN3
*Agrobacterium vitis* on grapevine rootstock cuts [28]	Static headspace analysis Evaluation of sensor drift	PEN3
*Erwinia amylovora* and *Pseudomonas syringae* pv. *syringae* on in vitro and dormant apple plants [20]	Lab to real scale VOCs concentration on adsorbents Dilution effects Three-class recognition	EOS507C PEN3
*Pseudomonas syringae* pv. *actinidiae* on in vitro kiwifruit plants [22]	Static headspace analysis at high RH	EOS507C PEN3
*Fusarium spp.* on wheat grain [29]	Static headspace analysis Air sample filtration on CaCO_3_	Prototype
*Anthonomus grandis grandis* on cotton bolls [30]	VOCs concentration on adsorbents Exclusion of water-sensitive sensors Dilution effects	Cyranose 320
Multiple pests on cucumber, pepper and tomato plants [31]	VOCs concentration on adsorbents	Bloodhound ST214
*Botrytis cinerea*, *Colletotrichum gloeosporioides* and *Alternaria sp.* on blueberry fruit [32]	Static headspace analysis Four-class recognition	Cyranose 320
*Aspergillus*, *Fusarium* and *Penicillium* spp. on oil palm [3]	Real scale	Cyranose 320
*Penicilium* spp. on orange fruit [33]	Static headspace analysis Dilution effects	LibraNose 2.1
*Botrytis*, *Fusarium* and *Penicillium* spp. on strawberry fruit [34]	Static headspace analysis External control of T and RH	PEN3
Multiple bacterial pathogens [35]	Static headspace analysis	PEN3
*Rhynchophorus ferrugineous* on ornamental palm [5]	8-day time course	PEN3
*Erwinia amylovora* on apple and pear plants; *Botrytis cinerea* and *Sclerotinia sclerotiorum* on kiwi fruit [8,36]	Static headspace analysis RH raised to 100%	EOS835
Multiple bacterial pathogens [37]	Static headspace analysis	EOS507C
*Ralstonia solanacearum and Clavibacter michiganensis* ssp. *sepedonicus* on potato tubers [38]	VOCs concentration on adsorbents Three-class recognition	Prototype
*Botrytis cinerea* on tomato plants [39]	Static headspace analysis Severity effects	PEN2
Multiple fungi and bacteria; *Ceratocystis fagacearum* on oak sapwood [11]	External control of RH	Aromascan A32S
*Nilaparvata lugens* [40]	Static headspace analysis	PEN2
*Rhyzopertha dominica* on wheat grain [41]	Static headspace analysis Dilution and time effects	PEN2
*Nilaparvata lugens* on rice plants [42]	Static headspace analysis Dilution effects	PEN2
Unclassified spider mites on cucumber; powdery mildew on tomato [43]	Static headspace analysis External control of T and RH	Bloodhound ST214

VOCs, volatile organic compounds.

**Table 2:** List of commercial e-nose models employed for plant pathogen and pest diagnosis.

E-Nose Model	Characteristics	Producer
AromaScan A32S	organic matrix-coated polymer-type 32-sensor array	Osmetech Inc., Wobum, MA, USA
LibraNose 2.1	quartz crystal microbalance 8-sensor array	Technobiochip, Pozzuoli, Italy
PEN2	Array of 10 metal-oxide semiconductor sensors (obsolete)	Airsense Analytics, Schwering, Germany
PEN3	Array of 10 metal-oxide semiconductor sensors, accumulation unit (EDU3)	Airsense Analytics, Schwering, Germany
EOS835	Array of 6 metal-oxide semiconductor sensors (obsolete)	Sacmi Scrl, Imola, Italy
EOS507C	Array of 6 metal-oxide semiconductor sensors, RH compensation system	Sacmi Scrl, Imola, Italy
Cyranose 320	Thin-film carbon-black polymer composite 32-sensor array	Smiths Detection Inc., Pasadena, CA, USA
Bloodhound ST214	14 organic polymer sensors (obsolete)	Scensive Technologies Ltd., Normanton, UK

**Table 3:** Performances of PEN3 e-nose equipped with different VOCs-sorbent cartridges (provided with electronic desorption unit, EDU3 (Airsense Analytics GmbH, Germany) in terms of ability to discriminate healthy from symptomatic potato tubers, infected by *Ralstonia solanacearum* or *Clavibacter michiganensis* ssp. *sepedonicus*. The percentage of symptomatic tubers, detected by conventional molecular diagnostic analysis (EU Directive 2006\56\CE) at the end of sensorial analysis, in the bulk sample is also reported (n.a. = not available).

VOCs-Sorbent Material	*Ralstonia solanacearum*		*Clavibacter michiganensis* ssp. *sepedonicus*	
	Symptomatic Tubers (%)	Discrimination Power * (%)	Symptomatic Tubers (%)	Discrimination Power * (%)
Tenax-TA	57	8	20	46
Carbotrap	67	23	0	25
Tenax-GR	57	0.5	n.a.	3
Carboxen	80	18	n.a.	46

* The discrimination power is a measure of non-overlapping between the healthy and diseased classes, and was calculated with Winmuster ver.1.6.2. (Airsense Analytics, Schwering, Germany).
